# Landscape of N1‐methyladenosin (m1A) modification pattern in colorectal cancer

**DOI:** 10.1002/cnr2.1965

**Published:** 2023-12-20

**Authors:** Chunhui Jiang, Yuan Tian, Chunjie Xu, Hao Zhang, Lei Gu

**Affiliations:** ^1^ Department of Gastrointestinal Surgery Renji Hospital, Shanghai Jiao Tong University School of Medicine Shanghai China

**Keywords:** colorectal cancer (CRC), N1‐methyladenosine (m1A) modification, prognosis, tumor immune microenvironment (TIME)

## Abstract

**Background:**

N1‐methyladenosine (m1A) is a recently identified mRNA modification. However, it is still unclear that how m1A alteration affects the development of colorectal cancer (CRC).

**Aims:**

The landscape of m1A modification patterns regarding tumor immune microenvironment (TIME) in CRC is a lack of knowledge. Thus, this study will utilize the public database to comprehensively evaluate of multiple m1A methylation regulators in CRC.

**Methods and results:**

We retrospectively analyzed 398 patients with CRC and 39 healthy people for negative control, using the The Cancer Genome Atlas (TCGA) database to evaluate m1A modification patterns regarding tumor immune microenvironment (TIME) in CRC. The m1Ascore was developed via principal component analysis. And its clinical value in prognosis of CRC was further explored. Our study revealed 12 key m1A‐related DEGs including CLDN3, MUC2 and CCDC85B which are identified associated with invasion and metastasis in CRC. The most important biological processes linked to weak immune response and poor prognosis were the regulation of RNA metabolism and RNA biosynthesis. Furthermore, we found that compared to patients with low m1A scores, those with high m1A scores had higher percentage, larger tumor burdens, and worse prognosis.

**Conclusion:**

Significantly diverse m1A modification patterns can be seen in CRC. Through its impact on TIME and immunological dysfunction, the heterogeneity of m1A alteration patterns influences the prognosis of CRC. This study provided novel insights into the m1A modification in CRC which might promote the development of personalized immunotherapy strategies.

## 1 INTRODUCTION

Colorectal cancer (CRC) is the world's fourth most fatal malignancy, responsible for almost 900 000 deaths per year. The incidence of colorectal cancer is predicted to increase to 2.5 million new cases in 2035.^1^ Currently, chromosomal instability, DNA replication errors, aberrant hypermethylation, and gene silencing are all potential pathways for CRC carcinogenesis.^2^ Each patient is genetically and epigenetically unique, confirmed via recent genome‐targeting investigations.^3^ Treatments for CRC include surgery, radiotherapy, chemotherapy, targeted therapy, and immunotherapy. Although the developed treatment options have doubled the overall survival of patients with CRC at advanced stage to 3 years, survival of those at early stage is still the highest. It is well known that different kinds of immune cells will impact tumor progression in any cancer types. TIME (tumor immune microenvironment) is a part of tumor microenvironment (TME) which is defined as various cell types including immune cells, fibroblasts, endothelial cells, etc. and extracellular components, such as growth factors, cytokines, extracellular matrix, hormones, surrounded by cancerous cells. TIME should be considered as an influencing factor in CRC development, as it has been shown to have a major impact on therapy response and clinical outcome.^4^

Recently, it has been revealed that m1A modification alteration is widely involved in the onset and progression of various diseases, including certain malignancies.^5^, ^6^ RNA methylation modifications N1 methyladenosine (m1A) is an important posttranscriptional modification of RNA which can be formed by adding a methyl group to the N1 position of adenosine. Multiple biological processes are affected by m1A dysregulation, including RNA structural stability, protein interactions, cell proliferation, and cell death.^7^, ^8^ The majority of m1A is found in a GC‐rich sequence with highly structured 5′‐untranslated regions (UTRs) close to the mRNA translation initiation site. Further, m1A methylation is regulated by a set of genes: “writers” (*TRMT6*, *TRMT10C*, *TRMT 61A* and *TRMT61B*),^9^, ^10^, ^11^ “readers” (*YTHDF1*, *YTHDF2*, *YTHDF3* and *YTHDC1)*,^12^ and “erasers” (*ALKBH1* and *ALKBH3*).^13^ “Writers” function as methyltransferase complexes, while “erasers” function as m1A demethylases, and “readers” directly bind to the reading frame of RNA with m1A and mediate translation and destruction.

Advancement in molecular mechanisms and pathophysiological understanding of TIME in tumor progression will increase the array of treatment options for a promising prognosis of patients with CRC. However, the landscape of m1A modification patterns regarding TIME in CRC is a lack of knowledge. Thus, this study will utilize the public database to comprehensively evaluate of multiple m1A methylation regulators in CRC.

## 2 MATERIALS AND METHODS

### 2.1 Data processing and unsupervised consensus clustering

In February 2023, the TCGA (http://cancergenome.nih.gov/) was used to gather the mRNA expression data and associated clinical data of patients with CRC. 39 healthy people and 398 patients with CRC were included in the RNA‐seq transcriptome data (FPKM format).Via consensus clustering analysis, the patients with CRC were separated into several categories according to the expression of 10 m1A regulators, which was carried out by the R package ConsensusClusterPlus. The m1A clusters' principal component analysis (PCA) was carried out using the R programs limma and ggplot2. Kaplan–Meier survival analysis was used, and log‐rank analyses were used to assess the difference between the m1A clusters. The heatmap was created in R via the heatmap package.

### 2.2 TIME cell infiltration assessment and gene set variation analysis

First, we used CIBERSORT analysis to determine the fraction of infiltrating immune cells in patients with CRC of different expression profiles. The survminer package in R was used to calculate the best cut‐off values for gene expression. Second, we used the ESTIMATE algorithm to compare the degree of immune cell infiltration among different clusters. The immune and stromal scores were calculated, and the tumor purity score and fraction were projected as a result. Published sources^14^, ^15^ were used to obtain a gene collection of human immunological subtypes. Third, we used single‐sample gene set enrichment analysis (ssGSEA) to determine the relative abundance and activity levels of each immune cell type in the TIME of CRC.

We used gene set variation analysis (GSVA) in R to look for changes in physiological processes depending on m1A clusters or m1A score. The significance level was chosen at .05 with an adjusted *p* value.

### 2.3 Differentially expressed genes (DEGs) associated to m1A were identified and analyzed

Based on previously reported methods,^16^ DEGs among distinct m1A clusters were found. The adjusted *p* value of .001 was used as the significant criterion for DEG screening. The limma tool in R's empirical Bayesian technique was used to moderate the standard errors of the estimated log‐fold change.

Using the cluster Profiler and ggplpt2 packages in R, we ran GO and KEGG pathway enrichment analyses on DEGs discovered between clusters 5 and 6. When *p* value equals .01 was chosen as the cut‐off criterion.

### 2.4 Construction of the m1A score

As previously published,^17^ we used the m1A score to objectively examine the effect of m1A alteration patterns on individual CRC patients. First, we eliminated overlapping DEGs among the various m1A clusters. Second, for overlapping DEG analysis, we classified patients with CRC into multiple groups via consensus clustering analysis. Third, we used univariate COX regression analysis to estimate each DEG's prognosis. Finally, via PCA analysis, we calculated the m1A score by a formula, which is similar to the Genomic Grade Index (GGI)^18^: m1A score = (PC1i + PC2i), where i represents the expression of m1A phenotype‐related genes.

The maftools package in R was then used to undertake tumor mutation burden (TMB) analysis on patients with CRC in high and low m1A score clusters. Via Spearman correlation analysis and previously reported gene sets,^19^, ^20^ we estimated the gene expression correlation coefficient between the m1A score and known biological gene signatures.

### 2.5 Statistical analysis

We used R software (version 3.5.3, https://www.r-project.org/) and GraphPad Prism v6.0 for Mac (GraphPad; San Diego, CA, USA) for statistical analysis and visualization. When considering more than two groups, one‐way ANOVA and Kruskal‐Wallis tests were employed. Student's t‐test was used to quantify the statistical differences between two groups. The *p* value was always two‐sided, and a *p*‐value <.05 was statistically significant.

## 3 RESULTS

### 3.1 The importance of m1A RNA methylation regulators for CRC prognosis

**FIGURE 1:**
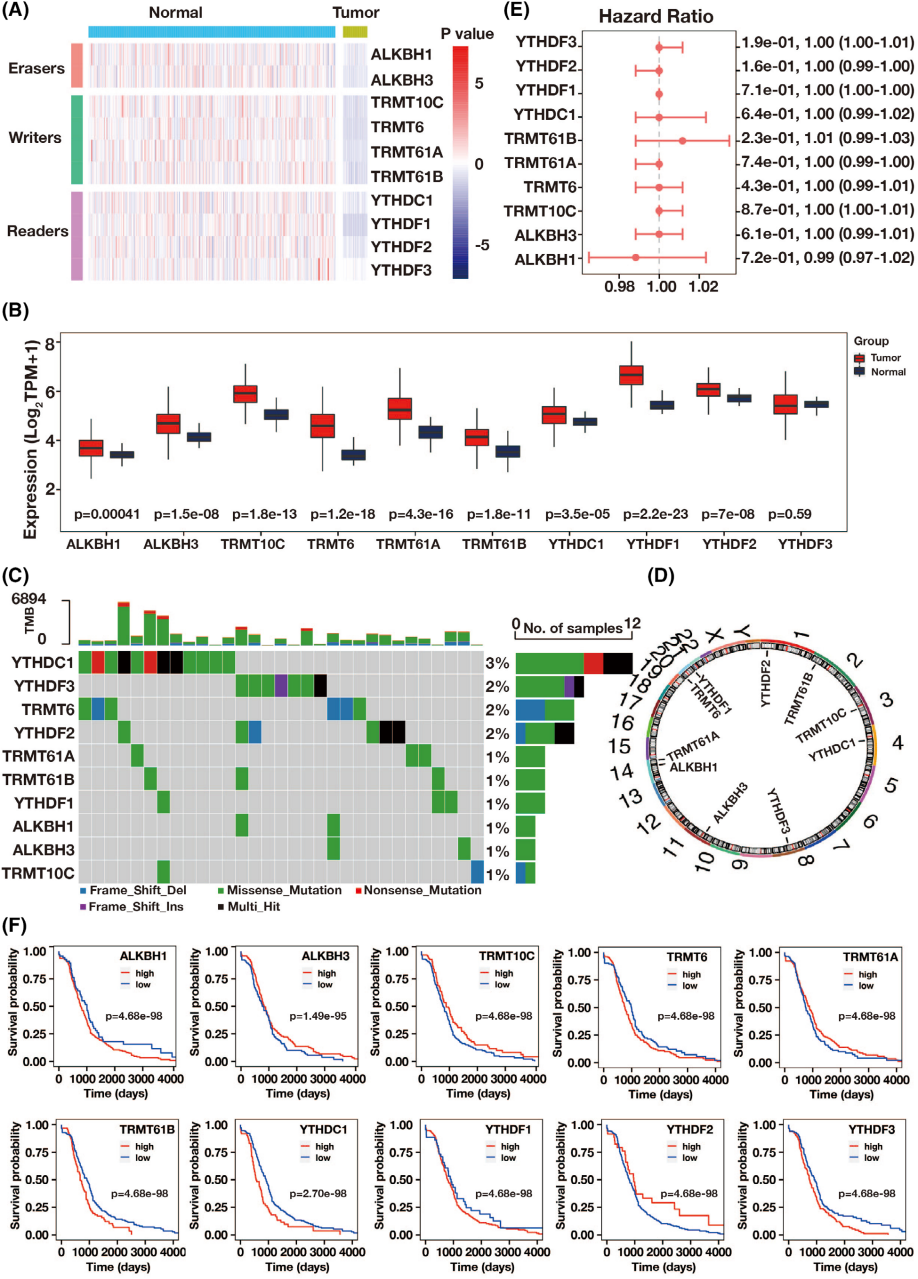
The expression variation and prognostic relevance of m1A regulators in CRC. (A) The expression levels of 10 m1A methylation modification regulators in each clinical sample. (B) The expression levels of 10 m1A methylation regulators in CRC samples and healthy controls. (C) The somatic mutations frequency and type of 10 m1A regulators. (D) The location of CNV alteration of m1A regulators was on different chromosomes. (E) Via a univariate Cox regression model, prognostic assessments for 10 m1A regulators were conducted. (F) M1A regulator survival analysis in patients with CRC.

Between CRC and normal tissue samples, there were differences of the expressional and genetic variants in m1A regulators. In this study, we examined 10 m1A regulators, including the “writers” TRMT10C, TRMT61B, TRMT6, and TRMT61A; the “readers” YTHDF1, YTHDF2, and YTHDC1; and the “erasers” ALKBH1 and ALKBH3. Compared to healthy controls, all m1A regulators except YTHDF3 were highly elevated in CRC samples (Figure 1A,B). 8.66% (31/358) of the samples contained multiple hits, nonsense mutations, missense mutations, frameshift mutations including deletion and insertion, and other mutation types. This somatic mutation frequency was very modest, with YTHDF2, YTHDF3, TRMT6, and YTHDC, which showed a comparatively greater frequency (Figure 1C). On many chromosomes, the m1A regulators' CNV modification was found (Figure 1D). We also looked into how well the m1A regulators predict prognosis of patients with CRC. In Figure 1E, the interactions between m1A regulators and their hazard function were depicted. All m1A regulators except TRMT61B and ALKBH1 indicated reduced risk and lack of relationship, respectively. The expression levels of all m1A regulators were related to the prognosis of CRC patients. High expression levels of m1A showed a significantly poorer chance of survival, while low expression levels of m1A showed a better chance of survival. (Figure 1F).

### 3.2 Consensus clustering of m1A regulators and estimation of immune microenvironment

**FIGURE 2:**
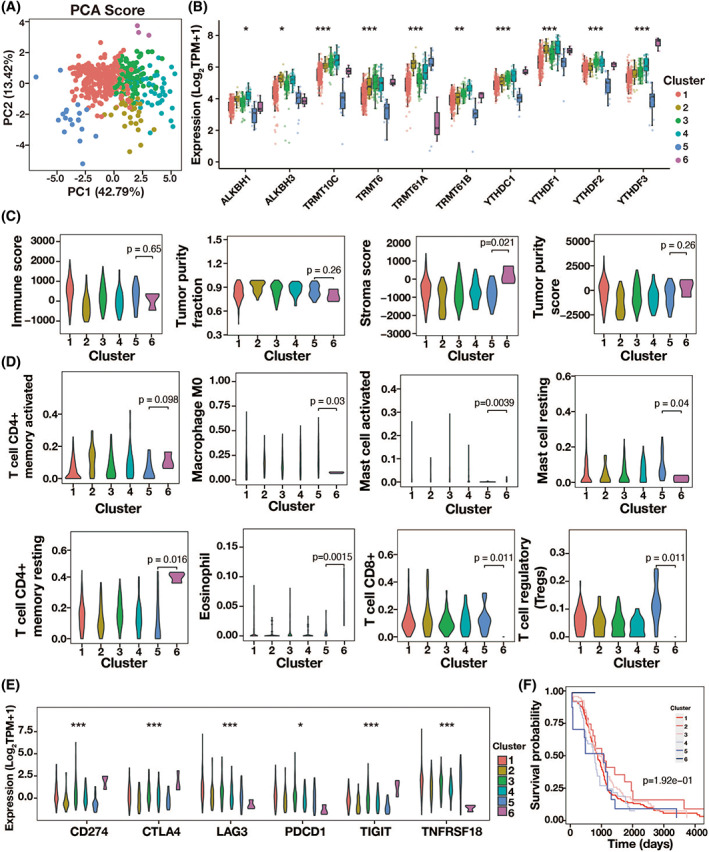
TIME related characteristics of 6 m1A modification clusters. (A) Principal component analysis. (B) The expression levels of 10 m1A methylation regulators among 6 m1A modification clusters. (C) Violin plots of the immune score, tumor purity faction, stromal score, and tumor purity score in 6 m1A modification clusters. (D) The fraction of tumor infiltrating lymphocyte cells in 6 m1A modification clusters. (E) The expression of immune checkpoint molecules in 6 m1A modification clusters. (F) The overall survival of patients with CRC in 6 m1A modification clusters. *p < .05; **p < .01; ***p < .001.

Based on the expression levels of m1A regulators, consensus clustering analysis separated patients with CRC into six groups. The PCA findings demonstrated that the six clusters were distinct from one another, particularly clusters 5 and 6 (Figure 2A). All of the m1A regulators' expression levels in cluster 6 were noticeably greater than those in cluster 5 (Figure 2B). After that, cluster 6 was found to have a much greater stromal score than the other clusters, but an investigation of immune cell infiltration revealed no significant differences in the immune and tumor purity score or tumor purity percentage across the six clusters (Figure 2C). Also, the enrichment of eight kind of immune cell types such as T cells, macrophages, and mast cells were remarkable different among m1A cluster1‐6, where cluster 6 had the lowest enrichment scores (Figure 2D). Additionally, cluster 6 had considerably decreased expression levels of immune checkpoint inhibitor (ICI) molecules like LAG3, PDCD1, and TNFRSF18 (Figure 2E). Finally, the Kaplan–Meier analysis demonstrated a significantly lower survival possibility in cluster 6 than in other clusters (Figure 2F). Therefore, m1A‐related patterns may affect the prognosis of patients with CRC which may associated with the changed immune microenvironment.

### 3.3 Biological analysis of m1A‐related DEGs

**FIGURE 3:**
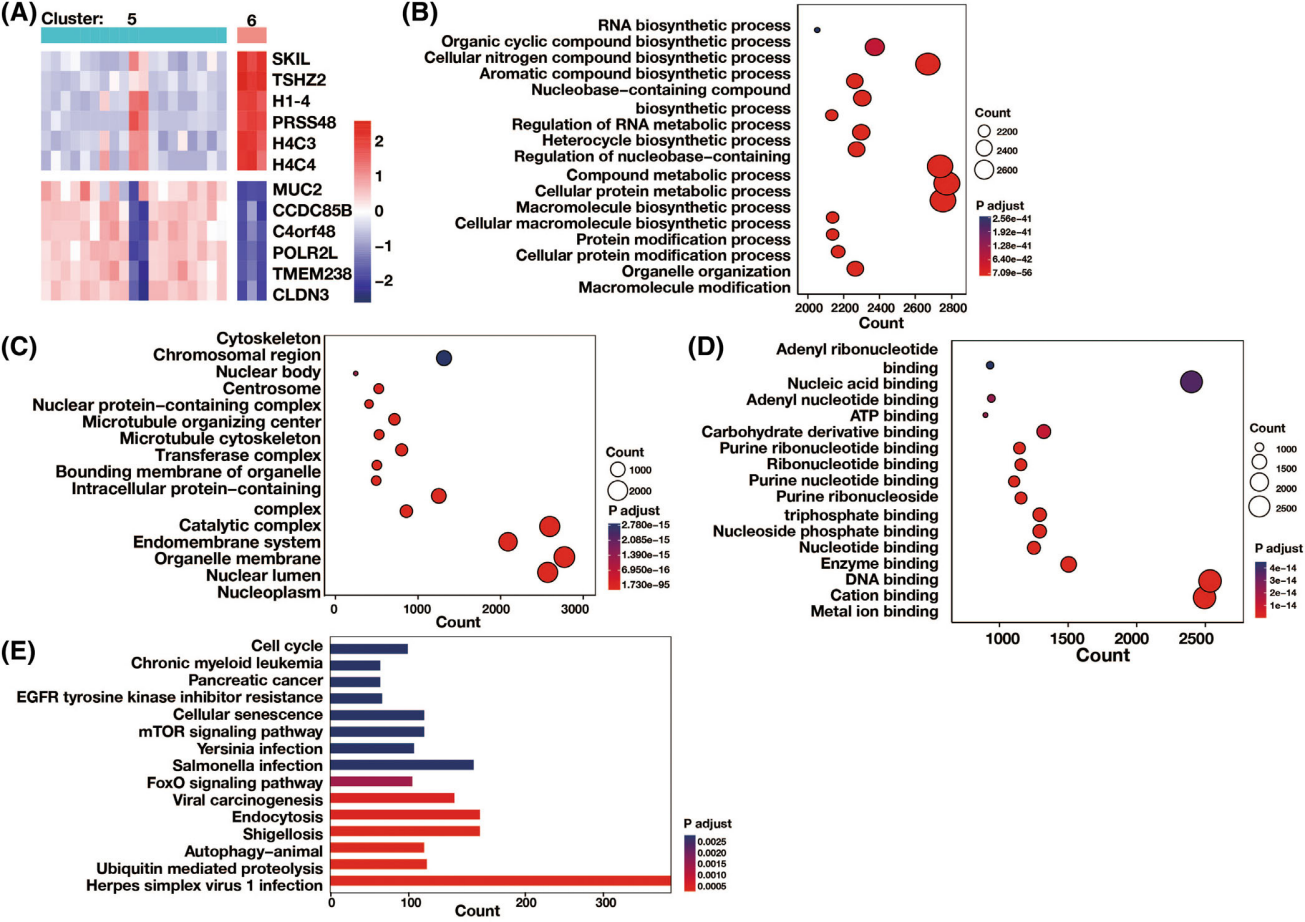
Compares the differentially expressed genes (DEGs) associated to m1A between m1A modification clusters 5 and 6. (A) Heat map of m1A‐related DEGs between cluster 5 and cluster 6. (B) The significant biological processes (BP) annotation for m1A‐related DEGs between cluster 5 and cluster 6. (C) Between clusters 5 and 6, there are strong cellular components (CC) annotations for DEGs connected to m1A. (D) The molecular functions (MF) annotation for m1A‐related DEGs between cluster 5 and 6. (E) The significant KEGG pathways for m1A‐related DEGs between cluster 5 and 6.

Because of the distinguished difference and clinical significance between cluster 6 and cluster 5, we chose them for further analysis. Between clusters 6 and 5, 6271 m1A‐related DEGs were filtered out, where the most significant DEGs were MUC2, CCDC85B, CLDN3, SKIL, TSHZ2, H1‐4, PRSS48, H4C3, H4C4, C4orf48, POLR2L, and TMEM238 (Figure 3A). For biological processes (BP) annotation, the m1A‐related DEGs were significantly enriched in RNA biosynthetic process, regulation of RNA metabolic process, regulation of nucleobase‐containing, etc. (Figure 3B). For cellular components (CC) annotation, the m1A‐related DEGs were remarkable involved in chromosomal region, centrosome, intracellular protein‐containing complex, etc. (Figure 3C). For molecular functions (MF) annotation, the m1A‐related DEGs were mainly localized in nucleic acid binding, adenyl nucleotide binding, DNA binding, etc. (Figure 3D). According to the KEGG database, the key KEGG pathways included EGFR tyrosine kinase inhibitor resistance, cellular senescence, mTOR signaling pathway, FoxO signaling pathway, and ubiquitin mediated proteolysis, among others (Figure 3E).

### 3.4 m1A score associated with CRC prognosis

**FIGURE 4:**
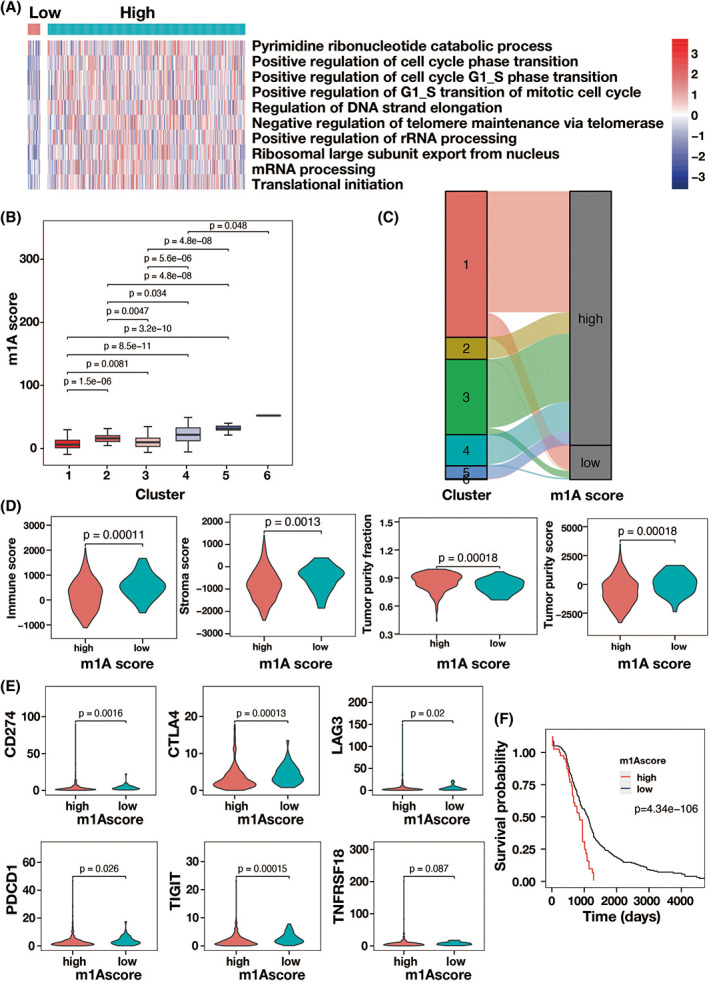
TIME related characteristics of high and low m1A score groups. (A) The heat map and the significant BP annotations between the low and high m1A score groups. (B) Different m1A score among 6 m1A modification clusters. (C) Alluvial diagram for different 6 m1A modification clusters and m1A scores. (D) Violin plots of the immune score, tumor purity faction, stromal score, and tumor purity score of high and low m1A score groups. (E) The fraction of tumor infiltrating lymphocyte cells of high and low m1A score groups. (F) The overall survival of patient with CRC of high and low m1A score groups.

To quantify m1A modification patterns, we divided individual patients with CRC into high and low m1A score groups via m1A score. The heat map showed the significant BP annotations between the two subtypes were enriched in pyrimidine ribonucleotide catabolic process, regulation of DNA strand elongation, mRNA processing, etc. (Figure 4A). There was also a significant different among six clusters, where cluster 6 exhibited the highest m1A score (Figure 4B,C). Compared with low m1A score group, the immune score and stromal score were found markedly higher in high m1A score group, and the tumor purity fraction and tumor purity score exhibit an opposite style via immune cell infiltration analysis (Figure 4D). Furthermore, the expression levels of CD274, CTLA4, LAG3, PDCD1, TIGIT, and TNFRSF18 were considerably lower in the high m1A score group than in the low m1A score group (Figure 4E). Finally, according to the Kaplan–Meier analysis, compared with low m1A score group, there was a significantly lower survival possibility in high m1A score group (Figure 4F).

**FIGURE 5:**
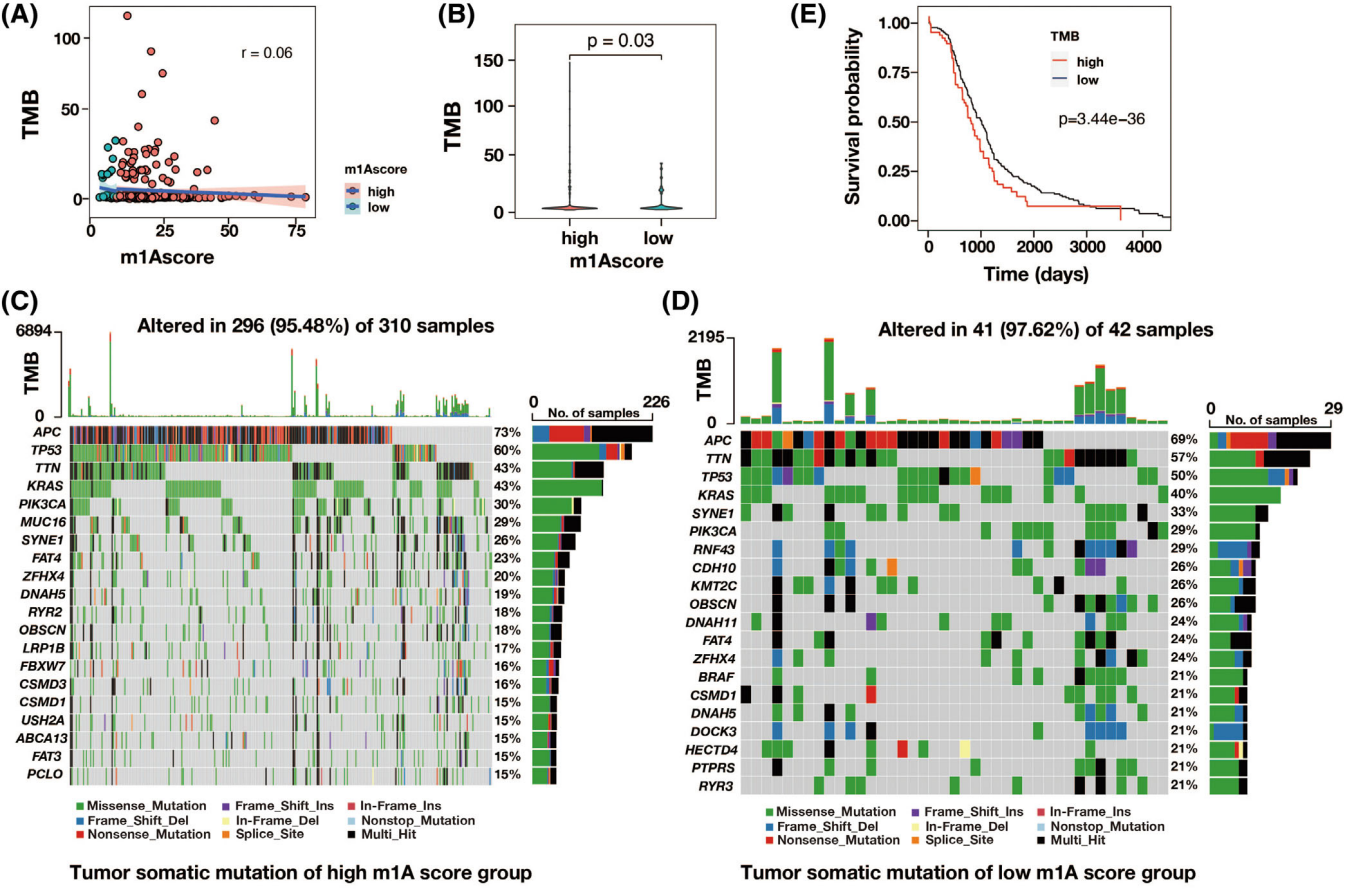
Tumor mutation related characteristics for high and low m1A score groups. (A) The correlation between m1A score and TMB. (B) A significant different TMB between high m1A score group and low m1A score group. (C) The landscape of major gene mutations in tumors from patients with CRC of high m1A scores. (D) In patients with CRC of low m1A scores, the landscape of major somatic mutations in the tumor. (E) The overall survival of patient with CRC of high and low TMB groups.

The link between m1A score and TMB was then confirmed via TMB quantification analysis. Tumors with a high m1A score were shown to be significantly positively connected with a greater TMB (*r* = 0.06, *p* = .03), as shown in Figure 5A,B. Furthermore, patients with CRC with high m1A scores had higher proportion of tumors and larger tumor burden than those with low m1A scores (Figure 5C,D). Finally, patients with CRC with elevated TMB had a worse chance of survival (*p* = .001) (Figure 5E). As a result, the m1A gene signature of patients with CRC was substantially linked with pathological advancement, which had a significant impact on the prognosis of CRC.

## 4 DISCUSSION

We studied m1A alteration patterns in CRC via a retrospective analysis of 398 patients with CRC and 39 normal people from the TCGA database. We discovered 12 key m1A‐related DEGs by analyzing the gene changes of the 10 m1A methylation regulators and their effect on TIME and prognosis of CRC. RNA biosynthetic process and regulation of RNA metabolic process were the most significant biological processes associated with low immune response and poor prognosis. Furthermore, we discovered that patients with CRC with high m1A scores had greater proportion, more extensive tumor burden, and a lower survival probability than those with low m1A values. This work deepened our understanding of m1A alteration in CRC, which could lead to the development of customized immunotherapy treatments.

By inhibiting Watson‐Crick base pairing and altering reverse transcription and protein translation, m1A modification can influence mRNA translation.^7^, ^8^ Here, compared with normal tissues, all m1A regulators expect *YTHDF3* were found significantly upregulating in CRC samples. Changes in CNV in m1A regulator genes might control mRNA expression levels and, as a result, affect the development of disease. Further, we found several mutation types in 8.66% CRC samples, with *YTHDC1* (3%), *YTHDF3* (2%), *TRMT6* (2%), and *YTHDF2* (2%) exhibiting a relatively higher frequency. Furthermore, we discovered that all m1A regulators were linked with the prognosis of patients with CRC, that is, the greater the expression levels of m1A regulators were, the lower the survival probability was. Furthermore, we discovered that patients with CRC with high m1A scores had greater proportion, more extensive tumor burden, and lower survival probability than those with low m1A values. As a result, we hypothesize that the variability of m1A alteration patterns in CRC will affect the prognosis of disease.

Based on 10 m1A regulators, we found six m1A alteration patterns. Cluster 6 was distinguished by the highest expression level of m1A regulators and, as a result, the highest stroma score. Also, the enrichment of eight kind of immune cell types including T cells, macrophages, and mast cells were remarkable low in cluster 6. Macrophages are an important component of the TIME, and they play an important role in tumor formation by secreting cytokines and chemokines and collaborating with inflammatory pathways. Research revealed that macrophages in CRC can switch phenotypical subtypes, promote tumor proliferation, invasion, and migration, and facilitate angiogenesis, mediate immunosuppression, regulate metabolism and interact with the microbiota.^21^ Several studies have revealed that mast cells release IL‐6, VEGF‐A, and C‐X‐C motif ligand 8 (CSCL8) and promote epithelial‐to‐mesenchymal transition in CRC cells under hypoxic conditions.^22^, ^23^ Additionally, there is a close relationship between mast cells and angiogenesis‐mediated tumor progression in CRC. High mast cells density is correlated with advanced stage, tumor progression and poor outcome in CRC.^24^, ^25^ Additionally, cluster 6 had significantly reduced expression levels for several ICI molecules, including LAG3, PDCD1, and TNFRSF18. Thus, the heterogeneity of m1A modification patterns impact the prognosis of CRC through its influence on TIME and immune dysfunction.

We identified 12 crucial DEGs for m1A, including CLDN3, MUC2, and CCDC85B. One of the primary components of tight junctions in the intestinal epithelium, claudin‐3 is encoded by CLDN3 and is essential for preserving cell–cell adhesion, barrier function, and epithelial polarity. Studies demonstrated that overexpression of claudin‐3 can increase the paracellular flux of macromolecules and the malignant potential of CRC cells. They concluded that the expression of *CLDN3* can be used to predict the prognosis of CRC.^26^, ^27^, ^28^ Von Willebrand factor (VWF) and MUC2 are believed to have shared a common ancestor.^29^ MUC2 is a secreted gel‐forming mucin which is the major structural component of the mucus in the colon.^30^ The prognostic significance of MUC2 in CRC is determined by invasion and metastasis, which are connected to MUC2 expression, according to the evidence.^31^, ^32^ Cyclin A2 and Cyclin B1, which act at the G2/M phase, are impacted by CCDC85B. The master regulator CCDC85B has been found to be able to change the cellular state of rapidly expanding subtype cells into slowly growing subtype cells in CRC. Its promise as a cutting‐edge treatment approach to stop the growth of malignant CRC has been suggested by in vitro studies.^33^ Furthermore, we found that the RNA biosynthetic, RNA metabolic, and mTOR signaling pathways were highly enriched in these m1A‐related DEGs. mTOR is a member of the phosphoinositide 3 kinase‐related kinase (PIKK) protein family. The phosphatidylinositol 3‐kinase (PI3K)/AKT pathway serves as the upper axis of the mTOR pathway. Accumulated evidence revealed the importance of the PI3K/AKT/mTOR pathway in tumorigenesis, proliferation, and progression in CRC.^34^, ^35^, ^36^

In conclusion, CRC exhibit significantly different m1A modification patterns. And the heterogeneity of m1A modification patterns impacts the prognosis of CRC through its influence on TIME and immune dysfunction. Additionally, this work revealed 12 key m1A‐related DEGs including *CLDN3*, *MUC2* and *CCDC85B* which are significantly associated with invasion and metastasis of CRC. This study provided insights into the comprehensive understanding of m1A modification in CRC which might promote the development of personalized immunotherapy strategies.

## AUTHOR CONTRIBUTIONS

**Chunhui Jiang:** Writing main manuscript; preparing figures. **Yuan Tian:** Data curation (equal); formal analysis (equal). **Chunjie Xu:** Validation (equal); visualization (equal). **Hao Zhang:** Validation (equal); visualization (equal). **Lei Gu:** Supervision (equal); writing – review and editing (equal).

## CONFLICT OF INTEREST STATEMENT

The authors have stated explicitly that there are no conflicts of interest in connection with this article.

## ETHICS STATEMENT

Approval of the research protocol by an Institutional Reviewer Board.

## Data Availability

All data and material in this study were available.
